# Increased Risk of Infection-Related and All-Cause Death in Hypercalcemic Patients Receiving Hemodialysis: The Q-Cohort Study

**DOI:** 10.1038/s41598-020-63334-8

**Published:** 2020-04-14

**Authors:** Shunsuke Yamada, Hokuto Arase, Masanori Tokumoto, Masatomo Taniguchi, Hisako Yoshida, Toshiaki Nakano, Kazuhiko Tsuruya, Takanari Kitazono

**Affiliations:** 1 Department of Medicine and Clinical Science, Graduate School of Medical Sciences, Kyushu University, Fukuoka, Japan; 2 Division of Internal Medicine, Fukuoka Dental College, Fukuoka, Japan; 3 Fukuoka Renal Clinic, Fukuoka, Japan; 4 Department of Medical Statistics, Osaka City University, Osaka, Japan; 5 Department of Nephrology, Nara Medical University, Nara, Japan

**Keywords:** Renal replacement therapy, Epidemiology

## Abstract

Although hypercalcemia is a risk factor for all-cause mortality in hemodialysis patients, it remains unknown whether hypercalcemia increases the risk of infection-related death. A total of 2869 hemodialysis patients registered in the Q-Cohort Study, a multicenter, prospective cohort study of hemodialysis patients, were analyzed. The predictor was albumin-corrected serum calcium level at baseline. The main outcome was infection-related death. Death risk were estimated by multivariable-adjusted Cox proportional hazard risk models and competing risk models. During the follow-up period of 4 years, 107 patients died of infection and 473 died of any cause. The patients were divided into four groups by the serum calcium level at baseline (G1, 5.7–8.9 mg/dL; G2, 9.0–9.4 mg/dL; G3, 9.5–9.9 mg/L; G4 10.0–16.5 mg/dL). In the multivariable-adjusted model, the incidence of infection-related death was significantly higher in the highest serum calcium group (G4) compared with the lowest serum calcium group (G1): hazard ratio [95% confidence interval], 2.34 [1.35–4.04], *P* = 0.002. Furthermore, higher serum calcium level was significantly associated with increased risk of all-cause death. In conclusion, our data suggest that a higher serum calcium level may be a risk factor for infection-related and all-cause death in hemodialysis patients.

## Introduction

Bone and mineral metabolism derangement, termed chronic kidney disease (CKD)-mineral and bone disorder (MBD), is highly prevalent in patients receiving hemodialysis^1^. CKD-MBD is often manifested as biochemical abnormalities, such as elevated serum levels of calcium, phosphate, and parathyroid hormone (PTH)^2^. Among these abnormalities, hypercalcemia is associated with increased risk of cardiovascular disease and all-cause mortality worldwide^3,4^. Mechanistically, hypercalcemia and calcium overload cause endothelial dysfunction and promote valvular and vascular calcification, which ultimately lead to increased incidence of cardiovascular events and death^5–7^. Thus, prevention of overt hypercalcemia and calcium overload in hemodialysis patients is an important therapeutic goal.

Infection is the second leading cause of death in hemodialysis patients^8,9^. Recent studies have shown that CKD-MBD is associated with infection-related death. For example, serum alkaline phosphatase level was associated with infection-related death in patients receiving peritoneal dialysis^10^. Meanwhile, PTH directly impaired leukocyte recruitment via PTH-1 receptor^11^. An *in vitro* study revealed that fibroblast growth factor 23 (FGF23), which is elevated in response to phosphate load, impaired leukocyte recruitment and was associated with an increased risk of infection-related hospitalization^12,13^. Taken together, CKD-MBD may increase the risk of infection and infection-related death in dialysis patients through disruption of host defense mechanisms. However, few studies have examined the direct association between calcium metabolism and infection or infection-related death in hemodialysis patients.

The present study aimed to determine whether serum calcium level is associated with risk of infection-related death in hemodialysis patients. To achieve this aim, we analyzed the dataset in the Q-Cohort Study, a multicenter, prospective, observational study of hemodialysis patients in Japan^14–16^.

## Materials and Methods

### Design of the Q-Cohort Study and study subjects

The Q-Cohort Study is a multicenter, prospective, longitudinal, observational study designed to identify risk factors for morbidity and mortality in patients undergoing maintenance hemodialysis. The details of the Q-Cohort study were reported previously^14–16^. Briefly, the study population consisted of 3598 outpatients aged ≥18 years who underwent regular hemodialysis therapy between December 2006 and December 2007 at 39 dialysis facilities in Fukuoka and Saga Prefectures in Japan. All patients were followed up until December 2010, unless they were lost to follow-up.

Among the 3598 patients registered in the study, 127 patients were excluded from the present analysis because of missing outcome data and 602 patients were excluded because of insufficient information on their baseline characteristics and medications. Thus, a total of 2869 patients were analyzed in the present study. The study was performed according to the Ethics of Clinical Research (Declaration of Helsinki). The study protocol was approved by the Kyushu University Hospital Institutional Review Board for Clinical Research (No. 20–31) and the study was registered in the University Hospital Medical Information Network clinical trials registry (UMIN000000556). All patients provided written informed consent prior to study participation.

### Definitions of outcomes and exposure

The primary outcome was infection-related death and the secondary outcome was all-cause death. Infection-related death was defined as death from any type of infection. The main exposure was albumin-corrected serum calcium level at baseline. The patients were divided into four groups (G1–G4) by the albumin-corrected serum calcium level at baseline: G1, 5.7–8.9 mg/dL; G2, 9.0–9.4 mg/dL; G3, 9.5–9.9 mg/L; G4 10.0–16.5 mg/dL. The albumin-corrected serum calcium level was calculated by Payne’s original formula as follows: albumin-corrected serum calcium level = serum albumin level (mg/dL) + 4 – serum albumin level (g/dL), if the serum albumin level was less than 4 g/dL^17^.

### Covariates and biochemical determinations

Baseline characteristics and potential confounding factors at baseline were collected by review of the medical records. Routine biochemical parameters were measured by auto-analyzers using standard procedures. Serum PTH level was measured by whole or intact PTH assays and the values measured by the two assays were interchanged with the following equation: intact PTH (pg/mL) = 1.7 × whole PTH (pg/mL). The target ranges during the follow-up period were as follows: albumin-corrected serum calcium, 8.4–10.0 mg/dL; serum phosphate, 3.5–6.0 mg/dL; serum intact PTH, 60–180 pg/mL^18^.

### Statistical analysis

Continuous variables and categorical variables were described as mean (standard deviation), median (interquartile range), or percentage. Baseline characteristics and laboratory data were compared among the four groups by trend analyses: Cochran–Armitage test for categorical variables and Jonckheere–Terpstra test for continuous variables.

Estimated risks of infection-related death and all-cause death among the four groups divided by albumin-corrected serum calcium level at baseline were calculated by Cox proportional hazard risk models. Adjustment for potential confounding factors was performed sequentially: unadjusted; Model 1 (age, sex); Model 2 (covariates in Model 1, presence of diabetic nephropathy, history of cardiovascular disease, dialysis vintage, dialysis time per session, dialysate calcium concentration, normalized protein catabolic rate, Kt/V for urea, serum levels of urea nitrogen, creatinine, total cholesterol, albumin, C-reactive protein, phosphate, alkaline phosphatase, and PTH, and use of vitamin D receptor activators (VDRAs) or calcium-based phosphate-binders). Adjusted hazard risks were expressed as hazard ratios (HRs) with 95% confidence intervals (95% CIs). When the association of albumin-corrected serum calcium level with all-cause mortality was examined, systolic blood pressure, cardiothoracic ratio, and blood hemoglobin level were also added as covariates in Model 2. After setting non-infection-related death as a competing risk, we determined the association between albumin-corrected serum calcium level and infection-related mortality using a Fine–Gray subdistribution hazards model. The adjusted hazard risk for every 1 mg/dL increase in albumin-corrected serum calcium level was also calculated with respect to infection-related death and all-cause death. To detect potential heterogeneity in the effects of albumin-corrected serum calcium level across baseline characteristics, a multiplicative interaction term was added to the relevant Cox regression model. A two-tailed *P*-value of <0.05 was considered statistically significant in all analyses. Statistical analyses were performed using JMP version 13.2 software (SAS Institute Inc., Tokyo, Japan) and R version 3.4.2 software (http://cran.rproject.org).

## Results

### Baseline characteristics of the participants

A total of 2869 patients were followed up for 4 years. The baseline characteristics of the patients in the four groups stratified by albumin-corrected serum calcium level are listed in Table 1. Patients with higher albumin-corrected serum calcium level showed lower prevalence of males, lower rate of diabetic nephropathy, longer median dialysis vintage, higher prevalence of 3.0 mEq/L dialysate calcium concentration, higher Kt/V for urea, higher body mass index, and lower systolic blood pressure level. Mean serum creatinine and phosphate levels were higher and mean serum albumin level was lower in patients with higher albumin-corrected serum calcium level. Patients with higher albumin-corrected serum calcium level showed higher frequency of intravenous VDRA administration and use of non-calcium-based phosphate binders than patients with lower albumin-corrected serum calcium level.

**Table 1:** Baseline clinical backgrounds of the patients in the four groups divided by the albumin-corrected serum calcium level at baseline (n = 2869).

	Four groups stratified by the baseline albumin-corrected serum calcium level				
	G1: 5.7–8.9 mg/dL	G2: 9.0–9.4: mg/dL	G3: 9.5–9.9 mg/dL	G4: 10.0–16.5 mg/dL	*P* for trend
	*n* = 787	*n* = 754	*n* = 703	*n* = 625	
**Baseline characteristics**					
Age, years	63.7 ± 13.0	63.8 ± 13.0	63.7 ± 12.7	62.9 ± 12.2	0.28
Sex (male), %	66	59	54	54	<0.001
Diabetic nephropathy, %	35	31	25	23	<0.001
History of cardiovascular diseases, %	32	32	36	30	0.11
Dialysis vintage, years	3.0 (0.9–7.4)	4.7 (1.7–9.5)	6.9 (3.3–13.1)	8.5 (3.8–15.3)	<0.001
Dialysis time per session, hours	4.7 ± 0.5	4.8 ± 0.5	4.7 ± 0.6	4.7 ± 0.6	0.01
Dialysate Ca concentration					
3.0 mEq/L, %	80	84	84	88	<0.001
2.5 mEq/L, %	20	16	16	12	<0.001
Kt/V for urea	1.53 ± 0.29	1.58 ± 0.30	1.62 ± 0.32	1.60 ± 0.30	<0.001
Normalized protein catabolic rate, g/kg/day	0.95 ± 0.21	0.96 ± 0.21	0.98 ± 0.21	0.96 ± 0.21	0.20
Body mass index, kg/m^2^	21.5 ± 3.4	21.2 ± 3.1	21.1 ± 3.1	20.9 ± 3.1	<0.001
Systolic blood pressure, mmHg	155 ± 23	155 ± 22	154 ± 24	153 ± 24	0.04
Cardiothoracic ratio, %	50.5 ± 5.7	50.4 ± 5.6	50.5 ± 5.4	50.9 ± 5.5	0.17
**Blood tests**					
Blood hemoglobin, g/dL	10.5 ± 1.2	10.5 ± 1.1	10.5 ± 1.1	10.6 ± 1.2	0.19
Serum albumin, g/dL	3.84 ± 0.37	3.83 ± 0.39	3.81 ± 0.42	3.75 ± 0.49	<0.001
Serum total cholesterol, mg/dL	151 (130–176)	156 (133–180)	153 (135–180)	151 (130–179)	0.42
Blood urea nitrogen, mg/dL	66.2 ± 15.2	65.8 ± 15.1	66.8 ± 14.8	65.5 ± 14.9	0.73
Serum creatinine, mg/dL	9.9 ± 2.7	10.2 ± 2.6	10.4 ± 2.6	10.7 ± 2.7	<0.001
Serum C-reactive protein, mg/dL	0.13 (0.01–0.32)	0.13 (0.06–0.30)	0.12 (0.05–0.30)	0.12 (0.04–0.30)	0.91
Albumin-corrected serum calcium, mg/dL	8.5 ± 0.4	9.2 ± 0.1	9.7 ± 0.1	10.4 ± 0.5	<0.001
Serum phosphate, mg/dL	4.9 ± 1.2	4.9 ± 1.2	5.0 ± 1.1	5.1 ± 1.2	<0.001
Serum alkaline phosphatase, U/L	247 (190–320)	232 (175–302)	231 (182–310)	227 (174–319)	0.53
Serum PTH (intact assay), pg/mL	126 (68–230)	99 (54–14)	89 (38–198)	95 (30–275)	0.11
**Medications**					
Use of anti-hypertensive drugs, %	64	63	63	59	0.11
Use of phosphate-binders, %	78	83	86	84	<0.001
Calcium-based phosphate-binders, %	71	76	7	67	0.11
Non-calcium-based phosphate-binders, %	24	24	32	38	<0.001
Use of VDRAs					
Oral administration, %	61	63	58	46	<0.001
Intravenous administration, %	7	9	15	29	<0.001

Baseline data are expressed as mean ± standard deviation, median (interquartile range), or percentage. The Cochran–Armitage test was used to determine P-values for trends of categorical variables and the Jonckheere–Terpstra test was used for continuous variables. A two-tailed P-value of <0.05 was considered statistically significant. PTH: parathyroid hormone; VDRAs: vitamin D receptor activators.

### Effects of serum calcium level on risk of infection-related death

**Figure 1:**
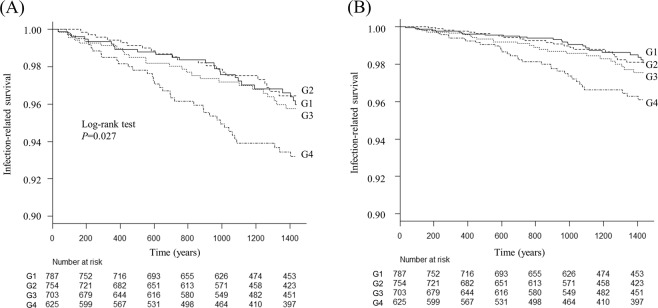
Kaplan–Meier curves for infection-related mortality stratified by four groups (G1–G4) divided by the albumin-corrected serum calcium level at baseline. (**A**) Non-adjusted curves. (**B**) Multivariable-adjusted curves. G1: 5.7–8.9 mg/dL; G2: 9.0–9.4 mg/dL; G3: 9.5–9.9 mg/L; G4: 10.0–16.5 mg/dL. The log-rank test was used to compare the non-adjusted survival curves among the four groups. The multivariable-adjusted curves were adjusted for baseline characteristics (age, sex, presence of diabetic nephropathy, history of cardiovascular events, dialysis vintage, dialysis time per session, serum levels of urea nitrogen, creatinine, albumin, C-reactive protein, phosphate, parathyroid hormone, alkaline phosphatase, and use of vitamin D receptor activators or calcium-based phosphate-binders). A two-tailed *P*-value of <0.05 was considered statistically significant.

During the median follow-up period of 4 years, 107 patients died of infection (G1: n = 25; G2: n = 21; G3: n = 25; G4: n = 36). Unadjusted and multivariable-adjusted Kaplan–Meier curves showed a significantly higher rate of infection-related death in the G4 group compared with the G1 group (log-rank test: *P* < 0.001) (Fig. 1). Unadjusted and multivariable-adjusted Cox proportional hazard risk analyses showed a significant association between higher serum calcium level and increased risk of infection-related mortality (HR [95% CI] in Model 2: 2.34 [1.35–4.04], *P* = 0.002) (Table 2).

**Table 2:** Association between albumin-corrected serum calcium level and the risk of infection-related mortality (n = 2869).

Models	Unadjusted model		*P* for trend	Model 1		*P* for trend	Model 2		*P* for trend
	HR (95% CI)	*P*-value		HR (95% CI)	*P*-value		HR (95% CI)	*P*-value	
Groups divided by the albumin-corrected serum calcium level at baseline									
G1; 5.7–8.9 (mg/dL)	1 (reference)			1 (reference)			1 (reference)		
G2; 9.0–9.4 (mg/dL)	0.89 (0.50–1.59)	0.70	0.015	0.93 (0.52–1.67)	0.87	0.003	0.99 (0.55–1.80)	0.98	0.001
G3; 9.5–9.9 (mg/dL)	1.09 (0.63–1.91)	0.51		1.21 (0.69–2.11)	0.51		1.39 (0.78–2.47)	0.27	
G4; 10.0–16.5 (mg/dL)	1.82 (1.09–3.03)	0.02		2.19 (1.31–3.66)	0.003		2.34 (1.35–4.04)	0.002	
Every 1 mg/dL increase in albumin-corrected serum calcium level	1.47 (1.18–1.84)	<0.001		1.54 (1.25–1.90)	<0.001		1.51 (1.20–1.89)	<0.001	

Serum calcium level was adjusted for serum albumin level using Payne’s formula as follows: albumin-corrected serum calcium level = serum calcium level (mg/dL) + 4 − serum albumin level (g/dL), if the serum albumin level was below 4 g/dL. The risk estimates are expressed as HR (95% CI). The HRs were estimated by the Cox proportional hazard risk model using a conventional approach. The following covariates were included in each model: Model 1, age, sex; Model 2, covariates in Model 1 and presence of diabetic nephropathy, dialysis vintage, dialysis time per session, dialysate calcium concentration, KT/V for urea, normalized protein catabolic rate, serum levels of urea nitrogen, creatinine, albumin, C-reactive protein, total cholesterol, phosphate, alkaline phosphatase, and parathyroid hormone, and use of vitamin D receptor activators and calcium-based phosphate-binders. A two-tailed *P*-value of <0.05 was considered statistically significant. CI: confidence interval; HR: hazard ratio.

**Figure 2:**
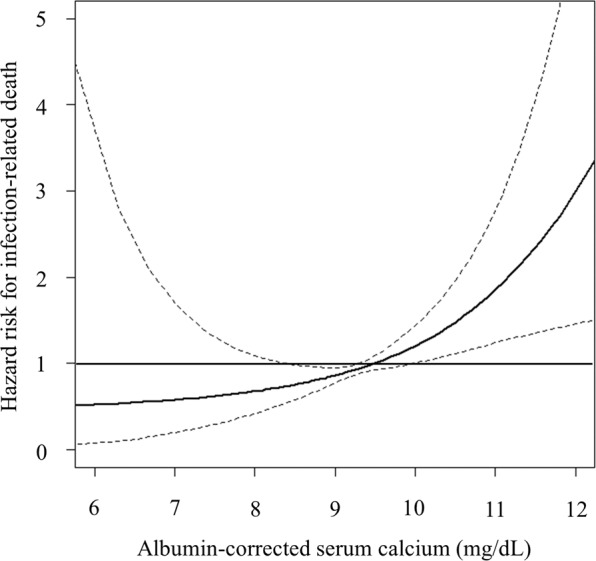
Multivariable-adjusted spline plots of hazard ratios and 95% confidence intervals for infection-related death according to albumin-corrected serum calcium level. Solid line: hazard ratios; dotted lines: 95% confidence intervals. The multivariable-adjusted Cox proportional hazard risk model was adjusted for age, sex, presence of diabetic nephropathy, history of cardiovascular disease, dialysis vintage, dialysis time per session, dialysate calcium concentration, normalized protein catabolic rate, Kt/V for urea, serum levels of urea nitrogen, creatinine, total cholesterol, albumin, C-reactive protein, phosphate, alkaline phosphatase, and PTH, and use of vitamin D receptor activators and calcium-based phosphate-binders.

When albumin-corrected serum calcium level was set as a continuous variable, higher albumin-corrected serum calcium level was significantly associated with increased risk of infection-related death (HR [95% CI] for every 1 mg/dL increase in albumin-corrected serum calcium level: 1.51 [1.20–1.89], *P* < 0.001). Spline curve analysis also showed that albumin-corrected serum calcium level was incrementally and significantly associated with increased risk of infection-related death, especially when the albumin-corrected serum calcium level exceeded 10 mg/dL (Fig. 2).

### Effect modifications by baseline characteristics regarding association between serum calcium level and incidence of infection-related death

**Figure 3:**
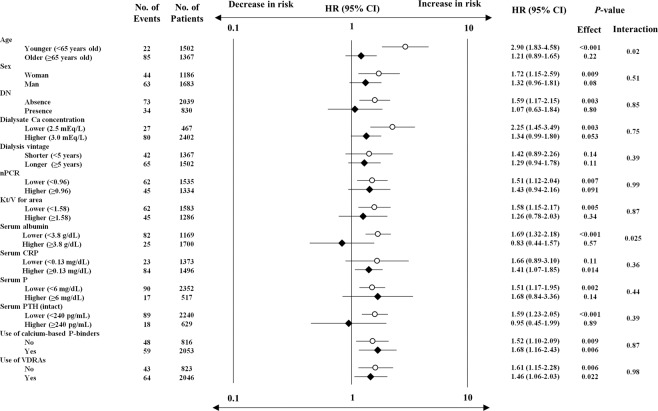
Multivariable-adjusted HRs and 95% CIs for incidence of infection-related death by every 1 mg/dL increase in albumin-corrected serum calcium level in subgroups of baseline characteristics. Open circles and filled rhombuses: point estimates of HRs; error bars: 95% CIs. The results were based on the final selected model. Variables relevant to the subgroups were excluded from each model. A two-tailed *P*-value of <0.05 was considered statistically significant. CI: confidence interval; CRP: C-reactive protein; DN: diabetic nephropathy; HR: hazard ratio; P: phosphate; PTH: parathyroid hormone; VDRAs, vitamin D receptor activators.

We determined the effect modifications in subgroups stratified by baseline characteristics for the association between every 1 mg/dL increase in albumin-corrected serum calcium level and incidence of infection-related death by multivariable Cox proportional hazard risk analysis. The association of albumin-corrected serum calcium level with infection-related death was significantly enhanced in younger patients and in patients with lower serum albumin level (Fig. 3). The other baseline characteristics showed no significant interactions with albumin-corrected serum calcium level (*P* = 0.36–0.91) (Fig. 3).

### Association between serum calcium level and risk of all-cause death

During the median follow-up period of 4 years, 473 patients died of any cause (G1: n = 111; G2: n = 104; G3: n = 126; G4: n = 132). Unadjusted and multivariable-adjusted Kaplan–Meier analyses showed that the highest albumin-corrected serum calcium level group (G4) had a significantly higher rate of all-cause death than the lowest albumin-corrected serum calcium group (G1) (Supplementary data, Fig. S1). Unadjusted and multivariable-adjusted Cox proportional hazard risk analyses revealed that the risk of all-cause death in the G4 group was significantly higher than that in the G1 group (multivariable-adjusted HR [95% CI]: 1.94 [1.48–2.55], *P* < 0.001) (Table 3). When albumin-corrected serum calcium level was set as a continuous variable, higher albumin-corrected serum calcium level was linearly and significantly associated with increased risk of all-cause death (HR [95% CI] for every 1 mg/dL increase in albumin-corrected serum calcium level: 1.32 [1.18–1.48], *P* < 0.001). Spline curve analysis also showed that albumin-corrected serum calcium level was incrementally and significantly associated with increased risk of all-cause death, especially when the albumin-corrected serum calcium level exceeded 10 mg/dL (Supplementary data, Fig. S2).

**Table 3:** Association between albumin-corrected serum calcium level and the risk of all-cause mortality (n = 2869).

Models	Unadjusted model		*P* for trend	Model 1		*P* for trend	Model 2		*P* for trend
	HR (95% CI)	*P*-value		HR (95% CI)	*P*-value		HR (95% CI)	*P*-value	
Groups divided by the albumin-corrected serum calcium level at baseline									
G1; 5.7–8.9 (mg/dL)	1 (reference)			1 (reference)			1 (reference)		
G2; 9.0–9.4 (mg/dL)	0.99 (0.76–1.30)	0.97	<0.001	1.04 (0.80–1.36)	0.76	<0.001	1.12 (0.85–1.47)	0.41	<0.001
G3; 9.5–9.9 (mg/dL)	1.24 (0.96–1.60)	0.10		1.38 (1.06–1.78)	0.02		1.53 (1.17–1.99)	0.002	
G4; 10.0–16.5 (mg/dL)	1.50 (1.17–1.93)	0.002		1.80 (1.39–2.32)	<0.001		1.94 (1.48–2.55)	<0.001	
Every 1 mg/dL increase in albumin-corrected serum calcium level	1.26 (1.12–1.41)	<0.001		1.33 (1.19–1.49)	<0.001		1.32 (1.18–1.48)	<0.001	

Serum calcium level was adjusted for serum albumin level using Payne’s formula as follows: albumin-corrected serum calcium level = serum calcium level (mg/dL) + 4 − serum albumin level (g/dL), when the serum albumin level was below 4 g/dL. The risk estimates are expressed as HR (95% CI). The HRs were estimated by the Cox proportional hazard risk model using a conventional approach. The following covariates were included in each model: Model 1, age, sex; Model 2, covariates in Model 1 and presence of diabetic nephropathy, dialysis vintage, dialysis time per session, dialysate calcium concentration, KT/V for urea, normalized protein catabolic rate, systolic blood pressure, cardiothoracic rate, blood hemoglobin, serum levels of urea nitrogen, creatinine, albumin, C-reactive protein, total cholesterol, phosphate, alkaline phosphatase, and parathyroid hormone, and use of vitamin D receptor activators and calcium-based phosphate-binders. A two-tailed *P*-value of <0.05 was considered statistically significant. CI: confidence interval; HR: hazard ratio.

### Sensitivity analyses

We examined the association between albumin-corrected serum calcium level and risk of infection-related death by setting non-infection-related death as a competing risk. A Fine–Gray subdistribution hazards model showed that higher albumin-corrected serum calcium level was significantly associated with heightened risk of infection-related death (HR [95% CI] per every 1 mg/dL increase in albumin-corrected serum calcium level: 1.52 [1.21–1.90], *P* < 0.001).

## Discussion

In the present study, we first showed that higher albumin-corrected serum calcium level was significantly associated with increased risk of infection-related death in patients receiving hemodialysis. The association remained significant even when analyzed by a competing risk model. Importantly, the association of hypercalcemia with infection-related death was enhanced in patients with lower serum albumin level and in younger patients. Spline curve analysis revealed that higher albumin-corrected serum calcium level was associated with incremental increase in risk of infection-related death. Furthermore, as reported previously, higher albumin-corrected serum calcium level was significantly associated with increased risk of all-cause mortality^4^. The present results suggest that hypercalcemia, a manifestation of CKD-MBD, is an independent risk factor for infection-related and all-cause deaths in maintenance hemodialysis patients.

Hypercalcemia may increase the incidence of infection or accelerate infection-related fatality. A previous longitudinal study showed that hypercalcemia was a risk factor for sepsis in critically ill patients^19^. Multiple studies have shown that patients on calcium channel blocker therapy were more likely to survive than patients without calcium channel blocker therapy under critical illness conditions^20^. In an *in vitro* study, increased intracellular calcium concentration induced leukocyte dysfunction, and calcium channel blockers inhibited calcium-induced calcium release in the intracellular space, thereby normalizing leukocyte recruitment^21^. Moreover, higher calcium concentration in the extracellular space increased the intracellular calcium level via L-type calcium channels and triggered various unfavorable cellular responses^22–25^. Accordingly, it is reasonable to consider that hypercalcemia can weaken host defense mechanisms by inhibiting leukocyte recruitment and exacerbating the infection itself or infection-related fatality. Further basic and experimental studies are needed to reveal the precise mechanisms for the clinical association between hypercalcemia and increased risk of infection-related death in hemodialysis patients.

Another potential explanation for the association between serum albumin-corrected calcium level and infection-related death may be calcium-induced increases in circulating FGF23 and calciprotein particles (CPPs). In previous experimental studies, calcium overload and ionized calcium were shown to up-regulate FGF23 synthesis and secretion in bone^26,27^. Notably, leukocyte recruitment, which is crucial in infections including sepsis, was prevented by FGF23^12^. An *in vitro* study on cultured human monocytes revealed that FGF23 also suppressed synthesis of active vitamin D, which enhances host defense mechanisms in immune cells. Furthermore, FGF23 acted on hepatocytes and macrophages to secrete inflammatory cytokines^28^. In turn, inflammation leads to malnutrition, ultimately resulting in decreased host defense activity. Taken together, calcium-induced FGF23 elevation may increase the risk of infection-related death by affecting host defense mechanisms and inducing inflammation and malnutrition.

CPPs, nanoparticles composed of calcium, phosphate, fetuin, and other proteins, have been implicated in a variety of pathologies associated with calcium and phosphate derangement^29,30^. Because calcium overload was reported to increase secondary CPPs^31^, a more toxic form of CPPs^32^, hypercalcemic patients are considered to have higher levels of secondary CPPs. Notably, secondary CPPs were shown to induce secretion of inflammatory cytokines by vascular smooth muscle cells and immune cells^33–35^. Thus, hypercalcemia can cause inflammation and subsequent malnutrition by increasing the circulating levels of secondary CPPs, leading to a heightened risk of infection-related death.

Subgroup analysis showed that the association between increased albumin-corrected serum calcium level and heightened infection-related mortality was enhanced in patients with lower serum albumin level and in younger patients. Because malnutrition was shown to be a strong inducer and predictor of infection^36^, it is reasonable that hypercalcemic patients with lower albumin level were at heightened risk of infection than those without hypoalbuminemia. Regarding the effect modification by age, it is unclear why the association between hypercalcemia and increased risk of infection-related death was enhanced in younger patients. Because subgroup analysis potentially increases alpha error of the entire study, the results obtained by subgroup analysis should be cautiously interpreted. In this regard, further studies are necessary to confirm whether interactions exist between serum calcium level and serum albumin level or age regarding infection-related death in hemodialysis patients.

In previous studies, the clinical backgrounds of the hypercalcemic patients were heterogenous and the causes of hypercalcemia included excess use of calcium-based phosphate binders and/or VDRAs, dialysates with higher calcium concentration, secondary hyperparathyroidism, low bone turnover, immobilization, and medical conditions that increased serum levels of calcitriol and PTH^37,38^. Notably, hypercalcemic patients showed relatively lower levels of serum PTH and alkaline phosphatase and were more likely to be treated with 3.0 mEq/L calcium dialysate and intravenous VDRAs. These observations suggest that the hypercalcemia in the present study was partly induced by calcium overload with a view to controlling secondary hyperparathyroidism. In fact, our baseline data were collected between 2006 and 2007. Because cinacalcet hydrochloride did not become available in Japan until 2008, secondary hyperparathyroidism was only treated with VDRAs and higher dialysate calcium concentrations at the time of our baseline data collection. Thus, the present analysis did not take the use of cinacalcet into account. Furthermore, in the era of calcimimetics, the prevalence of hypercalcemia and the clinical backgrounds of patients with hypercalcemia may have been altered. Accordingly, our data should be interpreted cautiously based on the historical context described above. In addition, because VDRAs were shown to exert protective effects against infection-related death in hemodialysis patients^15^, our data suggest that the dose of VDRAs for treatment of secondary hyperparathyroidism should be adjusted cautiously to avoid induction of VDRA-related hypercalcemia.

In the present study, hypercalcemia was associated with increased risk of all-cause mortality. This observation was consistent with previous reports^3,4^. Other lines of evidence have shown that calcium overload and intracellular calcium signaling enhance phosphate-induced vascular calcification by increasing PiT-1 expression in vascular smooth muscle cells and accelerating formation of CPPs^39–42^. Hypercalcemia was also shown to increase the risk of atrial fibrillation and ventricular arrhythmia^43^. Because hypercalcemia increases the circulating FGF23 level, it is likely that hypercalcemia-induced elevation of circulating FGF23 level increased cardiovascular death by accelerating left ventricular hypertrophy^44^. Taken together, these calcium overload-related changes may lead to cardiovascular events and death, thereby increasing the incidence of all-cause mortality in hemodialysis patients.

The strengths of the present study should be emphasized. First, the sample size was relatively large. Second, baseline data and outcomes were prospectively collected. Third, reasonable statistical techniques were applied for the adjustment of the baseline characteristics including various serum biochemical parameters as covariates. However, several limitations should be noted in the present study. First, the nature of the study meant that we were unable to confirm causality between hypercalcemia and infection-related death or all-cause death. Second, we had no data on the incidence of infection, and only analyzed infection-related mortality. Therefore, it remains unclear whether hypercalcemia actually increased the incidence of infection, or just accelerated the risk of fatality due to infection. In addition, the rate of infection could be much higher than the incidence of infection-related death. Third, we measured serum total calcium level and did not measure serum ionized calcium level. A recent study showed that albumin-corrected serum calcium level is likely to underestimate potential hypercalcemia in hemodialysis^45^. In this regard, our study may be affected by this kind of misclassification. Fourth, we only measured serum calcium level at baseline and did not take the trajectory of the serum calcium level into account, leading to raise potential misclassification of the patients. Our present observation should be confirmed by time-average model or time-dependent model that can deal with the impact of dynamic changes in serum calcium concentration during the observation period on the outcomes. Fifth, we were unable to adjust types of hemodialysis and dialysis conditions such as dialysis membrane and blood flow rate. Because those factors are reported to affect the amount of intradialytic calcium elimination and alter serum calcium level, it is possible that they may alter the association between serum calcium level and the risk of infection-related death if they are included as covariates in the multivariable analysis. Sixth, we neither specified the cause of hypercalcemia nor exclude primary hyperparathyroidism, bone metastases, multiple myeloma, or other medical conditions that cause hypercalcemia, although most of the cases were probably caused by CKD-MBD. It is possible that the cause of hypercalcemia may modify the impact of hypercalcemia on the risk of infection-related death in hemodialysis patients. Finally, we could not completely deny the possibility that unmeasured and residual confounding factors, including serum FGF23 levels, might have biased the observed association between hypercalcemia and increased risk of infection-related and all-cause mortality. With all these limitations, we expect that the present observations can provide medical practitioners with valuable information for the management of CKD-MBD and increased mortality in hemodialysis patients.

In conclusion, our results suggest that higher serum calcium level was associated with elevated risk of infection-related death and all-cause death in patients undergoing maintenance hemodialysis. Further studies are needed to determine whether hypercalcemia impairs the host defense mechanism and increases the risk of infection and infection-related death. Until then, our observations should be interpreted with caution.

## Supplementary information

Supplementary information.

## Data Availability

The datasets generated during and/or analyzed during the current study are available from the corresponding author on reasonable request.
