# Supplementary material for: Increased Risk of Infection-Related and All-Cause Death in Hypercalcemic Patients Receiving Hemodialysis: The Q-Cohort Study
Source: Sci Rep. 2020 Apr 14;10:6327. doi: 10.1038/s41598-020-63334-8 (PMC7156468; doi:10.1038/s41598-020-63334-8)
Supplement: Supplementary file 1 — Supplementary information. [file 41598_2020_63334_MOESM1_ESM.docx]

SUPPLEMENTARY DATA ONLINE

Increased Risk of Infection-Related and All-Cause Death in Hypercalcemic Patients Receiving Hemodialysis: The Q-Cohort Study

Shunsuke Yamada, MD, PhD,^1^ Hokuto Arase, MD,^1^ Masanori Tokumoto, MD, PhD,^2^ Masatomo Taniguchi, MD, PhD,^3^ Hisako Yoshida, PhD,^4^ Toshiaki Nakano, MD, PhD,^1^ Kazuhiko Tsuruya, MD, PhD,^5^ and Takanari Kitazono, MD, PhD^1^

^1^Department of Medicine and Clinical Science, Graduate School of Medical Sciences, Kyushu University, Fukuoka, Japan

^2^Division of Internal Medicine, Fukuoka Dental College, Fukuoka, Japan

^3^Fukuoka Renal Clinic, Fukuoka, Japan

^4^Department of Medical Statistics, Osaka City University, Osaka, Japan

^5^Department of Nephrology, Nara Medical University, Nara, Japan

**Correspondence:** Toshiaki Nakano, MD, PhD, Department of Medicine and Clinical Science, Graduate School of Medical Sciences, Kyushu University, 3-1-1 Maidashi, Higashi-ku, Fukuoka 812-8582, Japan

Tel: +81-92-642-5843; Fax: +81-92-642-5846

E-mail: toshink@med.kyushu-u.ac.jp**SUPPLEMENTARY FIGUERS**


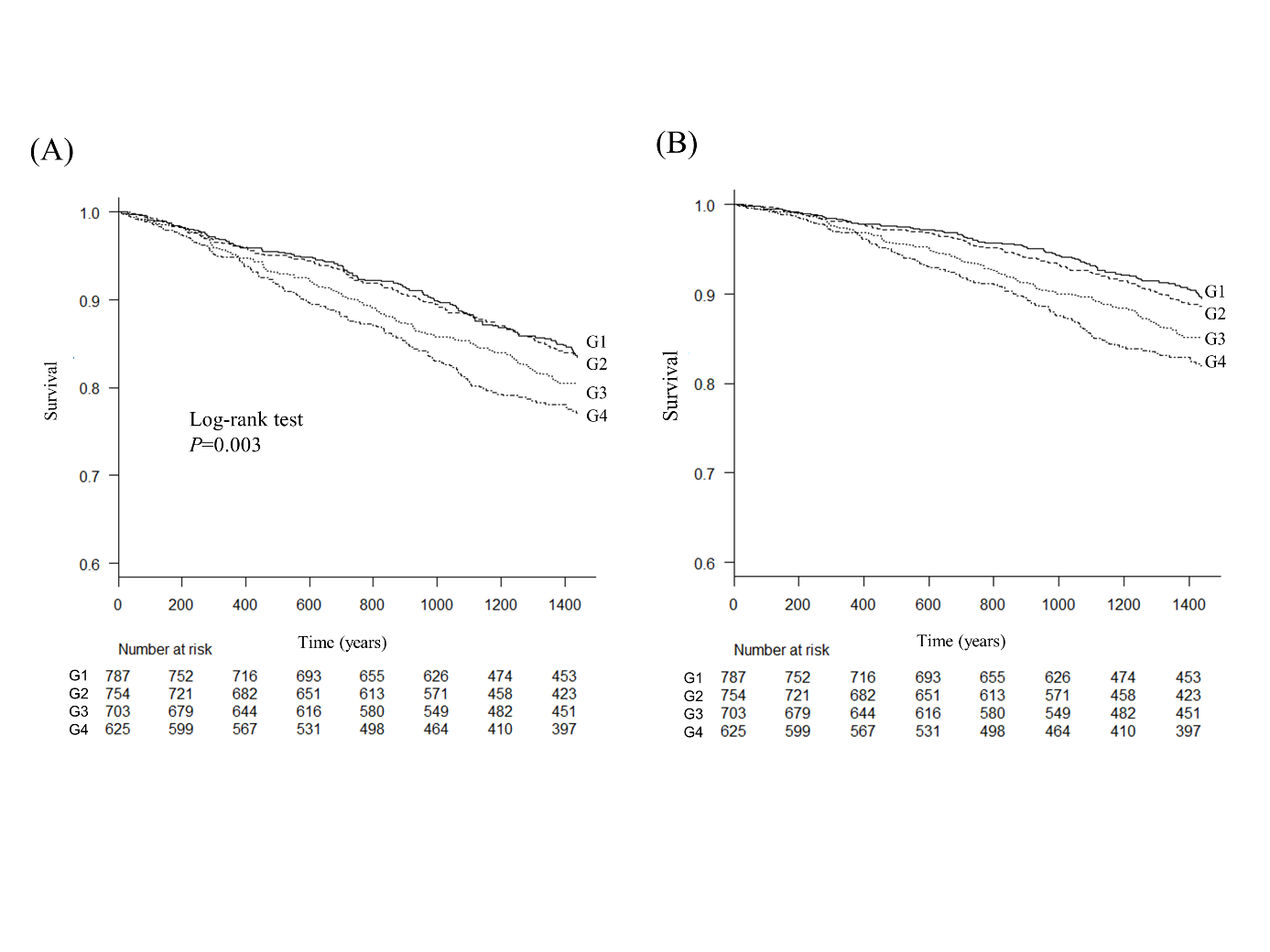


**Supplementary Fig. S1.** Kaplan–Meier curves for all-cause mortality stratified by four groups (G1–G4) divided by the albumin-corrected serum calcium level at baseline. (A) Non-adjusted curves. (B) Multivariable-adjusted curves. G1: 5.7–8.9 mg/dL; G2: 9.0–9.4 mg/dL; G3: 9.5–9.9 mg/L; G4: 10.0–16.5 mg/dL. The log-rank test was used to compare the non-adjusted survival curves among the four groups. The multivariable-adjusted curves were adjusted for baseline characteristics (age, sex, presence of diabetic nephropathy, history of cardiovascular diseases, dialysis vintage, dialysis time per session, dialysate calcium concentration, normalized protein catabolic rate, Kt/V for urea, systolic blood pressure, cardiothoracic ratio, blood hemoglobin, serum levels of urea nitrogen, creatinine, total cholesterol, albumin, C-reactive protein, phosphate, alkaline phosphatase, and PTH, and use of VDRAs and calcium-based phosphate-binders). A two-tailed *P*-value of <0.05 was considered statistically significant.


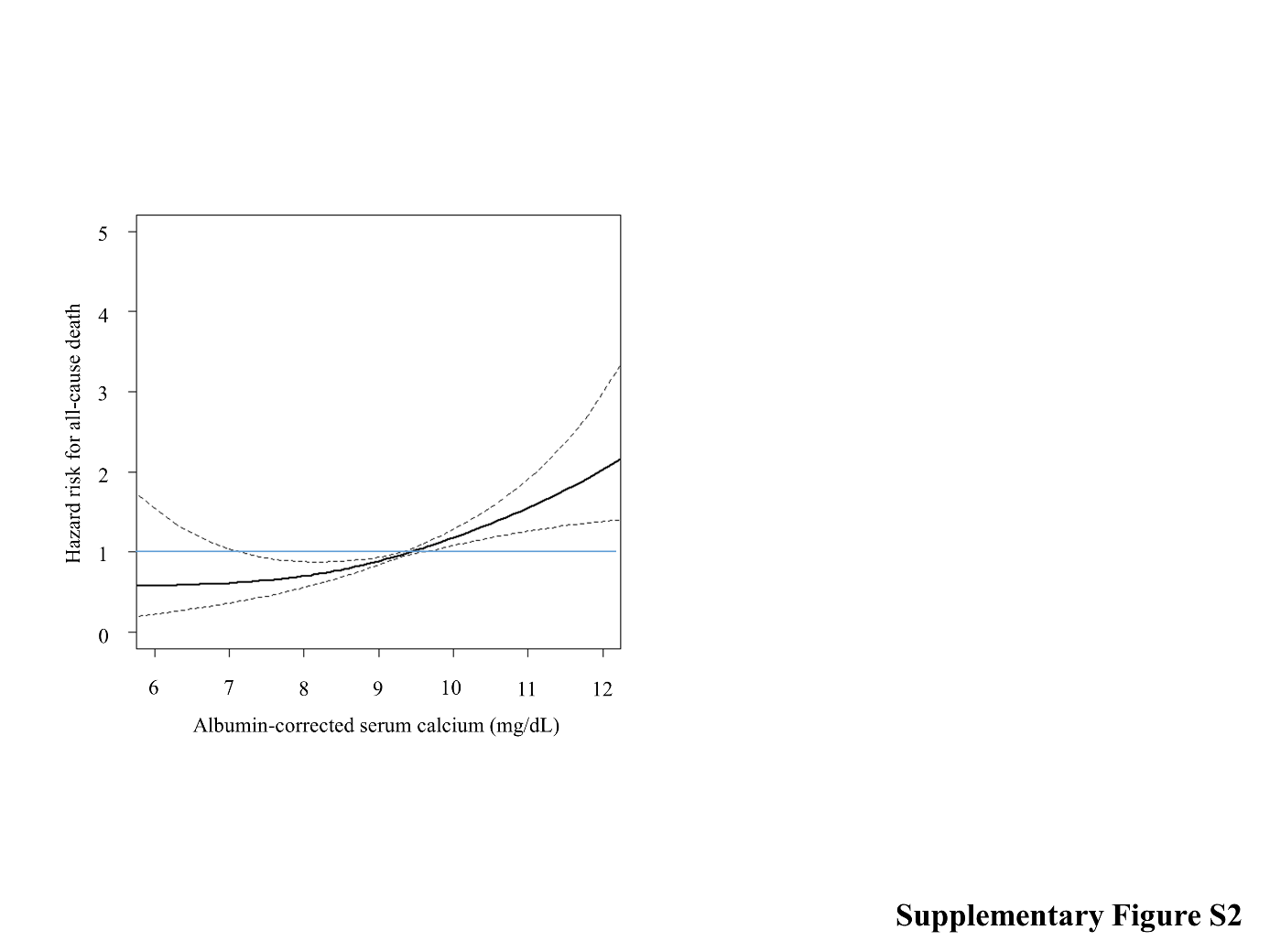


**Supplementary Fig. S2**. Multivariable-adjusted spline plots of hazard ratios for all-cause death according to albumin-corrected serum calcium level. Solid line: hazard ratios; dotted lines: 95% confidence intervals. The multivariable-adjusted Cox proportional hazard risk model was adjusted for age, sex, presence of diabetic nephropathy, history of cardiovascular diseases, dialysis vintage, dialysis time per session, dialysate calcium concentration, normalized protein catabolic rate, Kt/V for urea, systolic blood pressure, cardiothoracic ratio, blood hemoglobin, serum levels of urea nitrogen, creatinine, total cholesterol, albumin, C-reactive protein, phosphate, alkaline phosphatase, and PTH, and use of vitamin D receptor activators and calcium-based phosphate-binders.
